# Enrichment of HLA-DR^+^ neutrophils in osteoarthritic infrapatellar fat pad

**DOI:** 10.1016/j.isci.2026.115214

**Published:** 2026-03-05

**Authors:** Kajetana Bevc, Shipin Zhang, Andres Pazos, Ivan Berest, Marina Fonti, Gian Salzmann, Valentino Bruhin, Jakob Hax, Ana Alonso Perez, Rodolfo Gomez, Florian Mair, Isabelle C. Arnold, Marcy Zenobi Wong

**Affiliations:** 1 Tissue Engineering + Biofabrication Laboratory, Department of Health Sciences and Technology, ETH Zürich, Zurich, Switzerland; 2 Health Research Institute of Santiago de Compostela, Santiago Clinic Hospital, Santiago de Compostela, Spain; 3 Institute of Experimental Immunology, University of Zürich, Zurich, Switzerland; 4 Flow Cytometry Core Facility, ETH Zürich, Zurich, Switzerland; 5 Schulthess Klinik, Department for Knee Surgery, Zurich, Switzerland

**Keywords:** Immunology, Proteomics

## Abstract

Neutrophils are increasingly recognized as functionally versatile immune cells, with tissue-specific phenotypes that extend beyond their classical role in acute inflammation. In osteoarthritis (OA), neutrophils are abundant in synovial fluid (SF), which has been linked to worse symptoms. Here, we characterized neutrophils in OA IFP and identified a distinct population of HLA-DR^+^ neutrophils enriched in OA IFP. Sorted IFP HLA-DR^+^ neutrophils induced the proliferation of autologous CD3^+^ T cells, supporting their role in antigen presentation. Proteomic profiling of OA IFP revealed the upregulation of metabolic and enzymatic activities as well as the downregulation of apoptotic and enzyme inhibition pathways, consistent with the increased fibrosis and neovascularization observed histologically. Collectively, our findings expand the functional scope of neutrophils in arthritic diseases, further establishing their potential in antigen presentation, and position the IFP as a previously underappreciated, immunologically active niche in OA pathogenesis.

## Highlights

• OA IFP is more fibrotic with immune cell persistence
• Neutrophils are enriched in OA IFP compared to preOA IFP
• OA IFP neutrophils have an antigen-presenting HLADR+ CD74^+^ phenotype
• HLADR+ neutrophils from OA IFP stimulate T cell proliferation *in vitro*

## Graphical abstract

**Figure:**
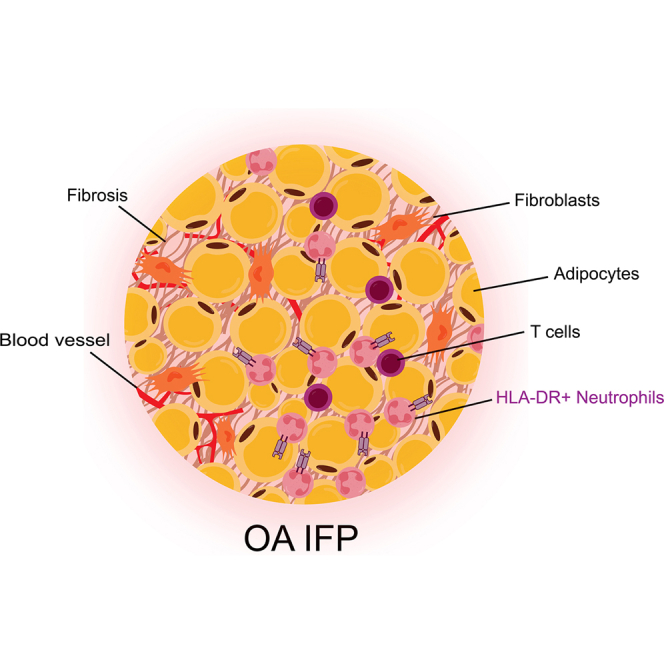

## Introduction

Neutrophils are the most abundant granulocytes and are recognized as rapid first responders of the innate immune response.^1^^,^^2^ They owe their abundance to continuous generation in the bone marrow, where they develop from proliferating myeloid progenitors. Their hallmark response is to degranulate and produce neutrophil extracellular traps (NETs) in response to a broad range of inflammatory stimuli. However, recently their plasticity and involvement in various homeostatic processes as well as disease mechanisms have been demonstrated.^1^^,^^2^^,^^3^^,^^4^ Interestingly, they populate most healthy organs,^5^ where they contribute to tissue stability and extracellular matrix (ECM) production.^6^ The notion that they are consistently short-lived cells has also been challenged as their half-life is reportedly longer in certain tissues.^2^^,^^7^ Therefore, neutrophils combine their capacity for organismal surveyance to contain the spread of pathogens with their remarkable functional plasticity that allows them to adjust to any microenvironment they are exposed to.^2^^,^^8^

Neutrophils also play a role in joint pathogenesis, as they are the first and often the most abundant immune cell type to enter the synovial space in different types of arthritis, including osteoarthritis (OA).^9^^,^^10^^,^^11^^,^^12^ OA is a chronic joint disorder characterized by an interplay of low-grade inflammation and mechanical damage, resulting in the progressive degeneration of joint tissues. While cartilage degradation has long been considered the hallmark of OA, other joint components, including synovial fluid (SF), synovial membrane, and increasingly infrapatellar fat pad (IFP) are recognized as key players in OA pathogenesis.^13^^,^^14^^,^^15^

The IFP is a fat deposit present in the knee joint that can act as a shock absorber and thus protects against joint damage.^16^^,^^17^ It is considered to be part of the same anatomo-functional unit as the synovial membrane^18^ and also plays a role in SF secretion and promoting joint lubrication.^16^ The volume of IFP of patients with OA has been found to be larger than healthy IFP, which has been positively correlated with clinical symptoms and pain.^13^^,^^17^^,^^19^ However, the volume changes of IFP in OA are still unclear, as there are studies reporting the opposite.^20^ It has also been well reported that OA IFP is highly fibrotic.^21^ Furthermore, OA IFP has an inflammatory phenotype and has been shown to secrete more inflammatory mediators, such as IL6, compared to IFP from patients with trauma and subcutaneous fat.^22^^,^^23^ OA IFP was found to be infiltrated by several immune cell types. Mast cells and both proinflammatory and anti-inflammatory/fibrotic M2-like (CD206^+^) macrophages are reportedly more abundant in OA IFP compared to non-OA IFP and subcutaneous fat.^22^^,^^24^^,^^25^ Clements et al. have shown a significant early increase of neutrophils in IFP in the rat monoiodoacetate model of OA.^26^ However, despite their suspected contribution to inflammation in OA SF, neutrophil function in human OA IFP remains largely unexplored, mostly due to technical challenges in isolating and analyzing these cells at the transcriptomic level.

Healthy SF contains few to no detectable cells, including neutrophils.^12^^,^^27^ In contrast, neutrophils make up roughly 26% of all cells in OA SF compared to 40–70% in healthy blood.^10^^,^^12^ A high neutrophil-to-lymphocyte ratio (NLR) in OA SF has also been shown to correspond to OA progression.^10^^,^^11^ IFN-γ response has been implicated as the driver of neutrophil infiltration into SF.^28^ This infiltration can have devastating consequences, as neutrophils secrete various cytokines and proteases such as neutrophil elastase (NE), matrix metalloproteinase 8 (MMP8) and MMP9.^12^^,^^29^^,^^30^ It has been shown that NE contributes to joint degeneration and can activate MMP13, an important enzyme in OA pathogenesis.^31^^,^^32^^,^^33^

In this study, we aimed to characterize the immune landscape of OA IFP, with particular emphasis on the under-explored role of neutrophils in this tissue.^34^^,^^35^^,^^36^^,^^37^^,^^38^

## Results

### OA IFP is more fibrotic, vascularized, and proteomically distinct from preOA IFP

**Figure 1:**
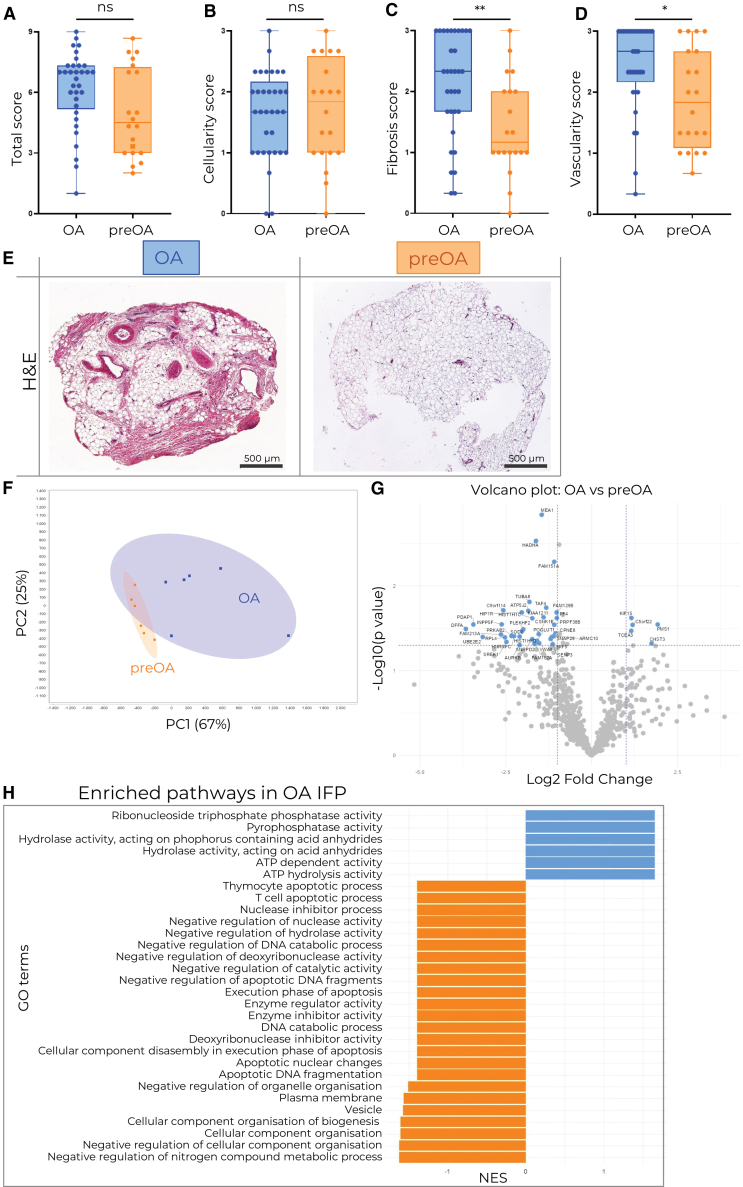
Analysis of IFP microenvironment Histological scoring of OA and preOA IFP (A–D) and representative H&E slides for each group (E). OA *n* = 33, preOA *n* = 20, Mann-Whitney test was used to assess statistical significance, OA = blue, preOA = orange, scale bar corresponds to 500 μm. (F–H) Proteomic analysis of OA and preOA samples (*n* = 6 per group). (F) Principal component analysis showing the disease-specific clusters (G). Differentially expressed proteins based on SWATH pathway enrichment analysis (H). Upregulated (blue) and downregulated (orange) in OA IFP are shown. Data is shown as a box and whisker plot (box: interquartile range, line: median, whiskers: minimum to maximum) with all individual data points displayed. Mann-Whitney test was used to assess statistical significance where ∗ = *p* < 0.05, ∗∗ = *p* < 0.01, ∗∗∗ = *p* < 0.001, and ∗∗∗∗ = *p* < 0.0001.

To evaluate the microenvironment of human IFP and uncover OA-specific changes, a total of 31 OA and 14 preOA IFP samples were collected (Table 1) and histologically scored (Table 2). We observed that, the OA IFP was significantly more fibrotic and vascularized than the preOA IFP (Figures 1C and 1D), although cellularity and total score did not significantly differ (Figures 1A and 1B). In addition, samples from both groups showed clear overall histological differences (Figure 1E). To further differentiate between the two disease states and to better understand their respective molecular composition, we performed a proteomic analysis of 6 OA and 6 preOA IFP samples. Principal component analysis indicated the segregation of the two groups into two distinct clusters, where OA IFP displays higher proteomic heterogeneity (Figure 1F). With the help of SWATH analysis, we found several proteins upregulated in OA (Figure 1G; Table S1). The most interesting in the OA context was carbohydrate sulfotransferase 3 (CHST3), as it is essential for normal cartilage development.^34^ More proteins were significantly downregulated in OA IFP (Figure 1G). Pathway enrichment analysis revealed significantly differentially expressed proteins between OA and preOA (Figure 1H). We show the upregulation of ATP hydrolysis activity, ATP-dependent activity, hydrolase activity acting on acid anhydrides and phosphate-containing anhydrides, as well as ribonuclease triphosphate phosphatase activity and pyrophosphatase activity (Figure 1H). We also observed the downregulation of apoptotic pathways, in particular T cell apoptotic processes and thymocyte apoptotic processes (Figure 1H). We also observed the downregulation of nuclease and deoxyribonuclease activity, enzyme inhibitor activity, cell component organisation/biogenesis, and negative regulation of catalytic activity in OA IFP compared to preOA IFP (Figure 1H).

**Table 1:** OA and preOA patient metadata

**Flow cytometry analysis OA vs. preOA IFP**							

Disease	Gender		Average Age	Average BMI	Average weight of IFP sample (g)	Average CD45^+^ Count	Average % of Live CD45^+^ cells (%)
	M	F					
OA	3	6	67.11	25.79	2.90	15025	64
preOA	3	5	38.50	26.17	0.97	3042	32

**Proteomic analysis OA vs. preOA IFP**						

Disease	Gender		Average Age	Average BMI	Average weight of analyzed IFP (mg)	Average Protein concentration (mg/mL)
	M	F				
OA	2	4	66.50	29.18	159.00	9.72
preOA	3	3	37.33	25.69	82.60	3.78

**single cell RNA sequencing of OA IFP**						

Tissue	Gender		Average Age	Average BMI	Average IFP sample weight (g) and SF volume (mL)	Total number of sequenced cells
	M	F				
IFP	2	5	70.57	30.14	2.79	1216
SF					5.91	1055

**Flow cytometry analysis OA IFP vs. SF**					

Tissue	Gender		Average Age	Average BMI	Average IFP sample weight (g) and SF volume (mL)
	M	F			
IFP	4	8	68.58	27.35	5.42
SF					3.47

**Coculture of HLA-DR+ Neutrophils with CD3^+^ T cells from OA IFP**					

Tissue	Gender		Average Age	Average BMI	Average IFP sample weight (g)
	M	F			
IFP	1	3	65.50	22.55	4.60

**Histology scoring of OA vs. preOA IFP**				

Disease	Gender		Average Age	Average BMI
	M	F		
OA	14	19	66.52	28.49
preOA	9	11	39.20	25.86

**Table 2:** IFP histological scoring protocol

Cellularity	0	Intact cellular connections
	1	Diffusion
	2	Severe diffusion
Fibrosis	0	Absent
	1	Mild (up to 20%)
	2	Moderate (20–40%)
	3	Severe (40+%)
Vascularity	0	No blood vessels
	1	1 to 2 blood vessels
	2	3 to 4 blood vessels
	3	5+ blood vessels

### Neutrophils and NK cells are enriched in OA IFP

**Figure 2:**
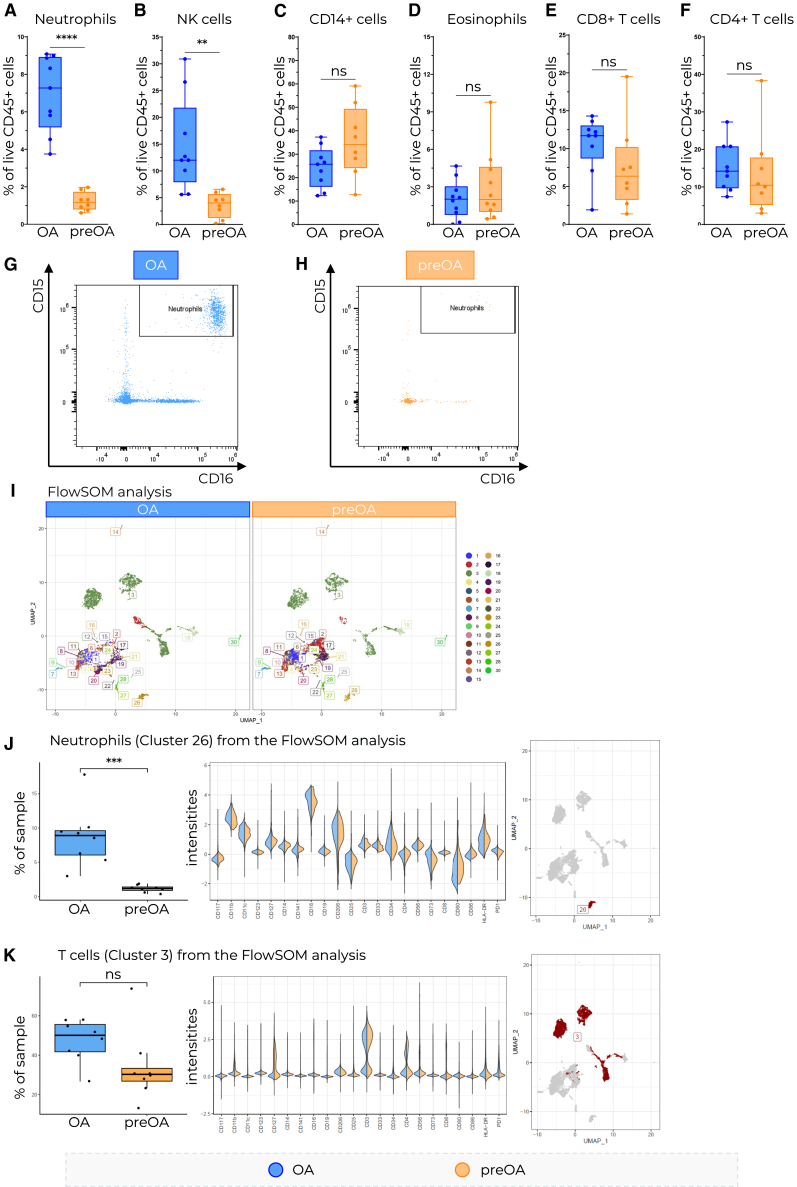
Characterization of IFP-infiltrating immune cells (A–H) Flow cytometry characterization of infiltrating immune cells based on manual gating. (I) FlowSOM cluster analysis and comparison between OA and preOA. (J–K) FlowSOM clusters 26 and 3 represent neutrophils and T cells. OA *n* = 9, preOA *n* = 8, Mann-Whitney test was used to assess statistical significance, OA = blue, preOA = orange. Data are shown as a box and whisker plot (box: interquartile range, line: median, whiskers: minimum to maximum) with all individual data points displayed. Unpaired *t* test was used to assess statistical significance where ∗ = *p* < 0.05, ∗∗ = *p* < 0.01, ∗∗∗ = *p* < 0.001, and ∗∗∗∗ = *p* < 0.0001.

To better understand the immune cell composition and its contribution to inflammation in the OA IFP, we isolated cells from 9 OA and 8 preOA IFP samples (Table 1) and analyzed them with spectral flow cytometry. Manual gating of the main immune cell lineages (Figure S3) revealed the enrichment of neutrophils and NK cell populations in OA IFP compared to preOA IFP (Figures 2A, 2B, 2G, and 2H). We further observed a trend toward CD14^+^ cell population enrichment in preOA IFP, while macrophage polarization markers (CD86, CD206) did not differ significantly between groups (Figure 2C; Figure S6). We did not observe any difference in eosinophil and T cell frequencies (Figures 2D–2F). To broadly evaluate differences between the sample groups in an unsupervised fashion, we performed an exploratory clustering with FlowSOM.^35^^,^^36^ IFP cells clustered into 30 distinct clusters (Figure 2I). According to neutrophil-specific marker expression, we identified cluster 26 as a neutrophil population. It was also the only cluster that was significantly enriched in OA IFP compared to preOA IFP (Figure 2J). Interestingly, this cluster exhibited higher HLA-DR expression relative to the HLA-DR intensity of other clusters (Figure 2J). Cluster 3, which we identified as T cells, showed a trend of enrichment in OA IFP (Figure 2K).

### HLA-DR+ neutrophils are enriched in OA IFP

**Figure 3:**
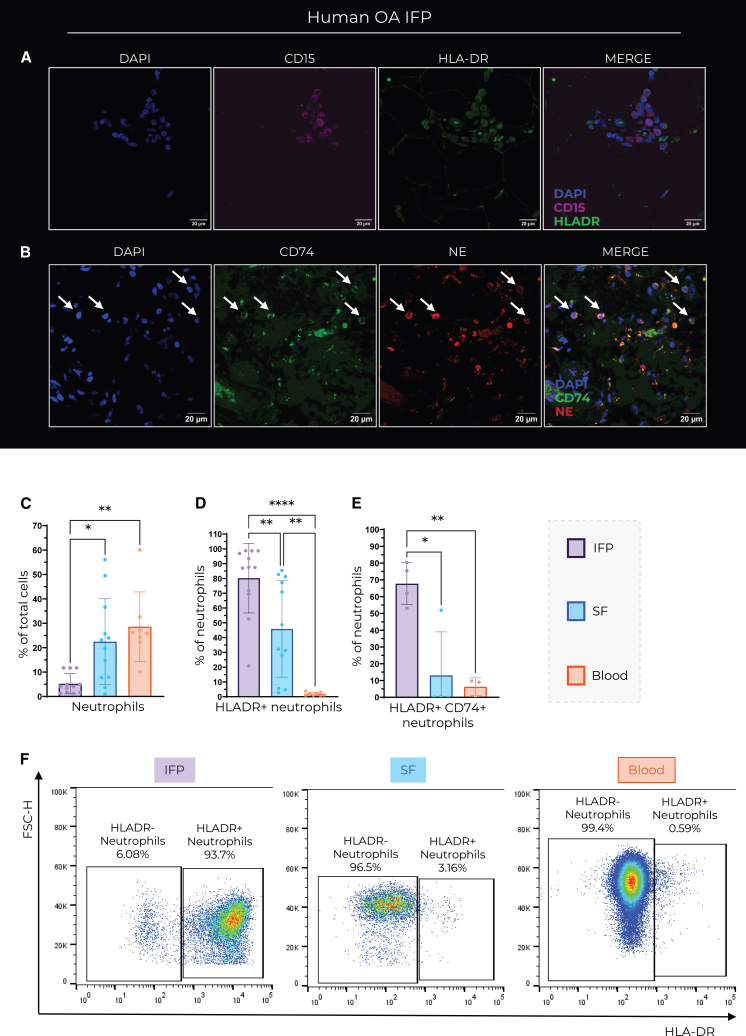
OA IFP Neutrophils express antigen-presenting markers (A and B) Immunofluorescent staining of OA IFP tissue sections shows the coexpression of (A) HLADR and CD15 and (B) CD74 and NE. Scale bars, 20 μm. (C–F) Flow cytometry analysis of (A) neutrophils IFP *n* = 12, SF *n* = 12, blood *n* = 8, and (B and F) HLADR+ neutrophils IFP *n* = 12, SF *n* = 12, blood *n* = 8, scale bars correspond to 20 μm. (E) HLADR+ CD74^+^ neutrophils IFP *n* = 4, SF *n* = 4, blood *n* = 3; in OA IFP = purple, OA SF = blue, and healthy blood = orange. Data are represented as mean ± SD. Ordinary one-way ANOVA was used to assess statistical significance where ∗ = *p* < 0.05, ∗∗ = *p* < 0.01, ∗∗∗ = *p* < 0.001, and ∗∗∗∗ = *p* < 0.0001.

To validate the expression of HLA-DR on neutrophils, we performed immunofluorescence staining of the human OA IFP with neutrophil-specific markers (CD15, NE) in combination with antigen presentation markers (HLA-DR, CD74). This analysis revealed cells co-expressing neutrophil and antigen presentation markers in OA IFP tissue sections (Figures 3A and 3B). Specifically, we found cells that co-express CD15 and HLA-DR (Figure 3D) as well as NE and CD74 (Figure 3E). To quantify how many neutrophils express HLA-DR, we analyzed them by flow cytometry. We compared the HLA-DR expression of neutrophils in SF and IFP of the same patients with OA, as well as to healthy blood donors. We show that there were significantly less neutrophils in OA IFP compared to healthy blood and matched SF; however, significantly more IFP neutrophils express HLA-DR (Figures 3C, 3D, and 3F). On average, 65% of neutrophils isolated from the OA IFP co-expressed HLA-DR with CD74, which is significantly more compared to matched SF as well as healthy blood (Figure 3E).

### HLA-DR+ neutrophils induce T cell proliferation *in vitro*

**Figure 4:**
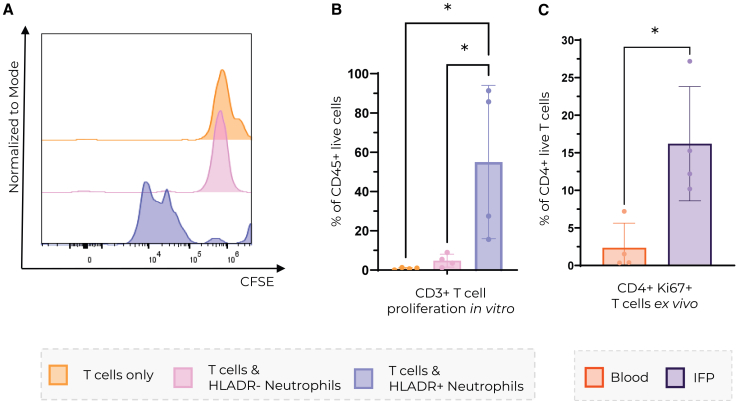
IFP HLADR+ neutrophils stimulate T cell proliferation *in vitro* (A) Histogram of T cell CFSE intensity and (B) quantification of T cell proliferation based on T cell CFSE intensity after monoculture = orange, coculture with autologous HLADR-neutrophils = pink, and autologous HLADR+ neutrophils = blue. Ordinary one-way ANOVA was used to assess statistical significance. (C) Quantification of Ki67 expression on CD4^+^ T cells *ex vivo*. *t* test was used to assess statistical significance. n per group = 4. Data are represented as mean ± SD, ∗ = *p* < 0.05, ∗∗ = *p* < 0.01, ∗∗∗ = *p* < 0.001, and ∗∗∗∗ = *p* < 0.0001.

To explore the functional phenotype of HLA-DR^+^ neutrophils, we sorted autologous CD3^+^ T cells and HLA-DR^+^ neutrophils from the OA IFP, along with CD3^+^ T cells and HLA-DR^-^ neutrophils from healthy donor blood. As neutrophils are fragile cells, we assessed neutrophil viability after sorting, which was above 98% (Table S3). We stained T cells with CFSE before co-culture to assess proliferation. T cells were co-cultured with autologous HLA-DR^+^ or HLA-DR^-^ neutrophils, without the addition of external stimuli or antigens. Specifically, we co-cultured CD3^+^ T cells with HLA-DR- neutrophils from the blood of the same donor and CD3^+^ T cells with HLA-DR+ from the same OA donor. Notably, T cells co-cultured with HLA-DR^+^ neutrophils showed significantly increased proliferation compared to T cells cultured alone (Figures 4A and 4B), indicating that HLA-DR^+^ neutrophils can functionally process and present antigens to T cells. To assess if this phenotype may be recapitulated *in vivo*, we compared the proliferation of T cells from the OA IFP and healthy blood upon staining with Ki67. Interestingly, a significantly higher proportion of Ki67^+^ T cells was observed in OA IFP compared to healthy blood (Figure 4C). It must be noted that the experiments were performed with an n of 4 due to the constraints pertaining to obtaining the patient samples. Combined with the proteomic data showing the downregulation of T cell apoptotic processes in the OA IFP, our results suggest that T cells survive longer in the OA IFP and proliferate, possibly due to stimulation by HLA-DR+ neutrophils (Figures 1H and 4A–4C).

Together, these results highlight a previously unrecognized immunoregulatory axis in the OA IFP, where tissue-adapted neutrophils may contribute to chronic inflammation by promoting T cell activation and persistence.

## Discussion

This study provides insights into the cellular and molecular landscape of the OA IFP, highlighting the enrichment of HLA-DR^+^ neutrophils. While previous studies have reported the involvement of neutrophils in OA disease progression, particularly in SF,^10^^,^^28^^,^^31^ our data indicate that neutrophils also accumulate in the human OA IFP (Figure 2A). Using a combination of histology, flow cytometry, proteomics, and *in vitro* experiments, we demonstrated that infiltrating neutrophils express HLA-DR and CD74, markers typically associated with antigen-presenting cells that activate CD4^+^ T cells.

Neutrophils are enriched in OA IFP compared to preOA IFP and they express HLA-DR at higher levels compared to SF neutrophils from the same OA donors (Figures 2A and 3C). Furthermore, these neutrophils expressed higher levels of HLA-DR (Figures 3D and 3E). This is further supported by immunofluorescence data showing HLA-DR^+^CD15^+^ and CD74^+^NE^+^ cells in OA IFP (Figures 3A and 3B). Even though neutrophils are not traditionally considered to be antigen-presenting cells, a number of studies reported their expression of antigen presentation markers in response to inflammatory stimuli^37^^,^^38^^,^^39^^,^^40^^,^^41^ - but not in peripheral blood under homeostatic conditions^37^^,^^42^ - which is consistent with our findings (Figures 3C and 3F). This suggests that the OA IFP microenvironment promotes neutrophil HLA-DR expression. However, the expression of HLA-DR does not mean that neutrophils can effectively present antigens to T cells. Antigen presentation to T cells usually leads to their activation and proliferation.^43^ Therefore, neutrophils expressing MHC II with a loaded antigen should be able to induce T cell proliferation. There is evidence that human neutrophils can indeed present tetanus toxoid to T cells and activate them without the presence of anti-CD3 or anti-CD28 in cell culture.^39^^,^^44^^,^^45^ In this study we provide data suggesting that *ex vivo* HLA-DR^+^ neutrophils sorted from the IFP may induce donor-matched CD3^+^ T cell proliferation (Figures 4A and 4B). Our findings add to a growing body of evidence that tissue-resident neutrophils can indeed gain antigen-presenting capabilities in response to local cues or prolonged residence.

OA is primarily driven by innate immune mechanisms, T cell infiltration has previously been reported in OA synovium.^46^^,^^47^^,^^48^ Specifically, CD4^+^ T helper cells have been implicated in OA pathogenesis and in crosstalk with innate immune cells to promote OA progression.^49^^,^^50^ Consistent with this, our data indicates T cells proliferate in OA IFP and that HLA-DR^+^ neutrophils in the OA IFP are positioned to interact with T cells, potentially facilitating local immune modulation (Figure S7).

Proteomic analysis of the IFP shows the upregulation of metabolic and enzymatic pathways in OA IFP, indicating elevated energy demand, metabolic flux, and potentially enhanced cell signaling. Upregulated ATP-driven processes suggest immune activation, degranulation, or ROS production. Furthermore, apoptotic pathways and reduced negative regulation of enzymatic processes are downregulated in OA IFP (Figure 1H). This suggests that cells, possibly T cells and other immune cells, may be surviving longer, which could contribute to chronic immune persistence and subsequent tissue damage. Notably, the enrichment of enzymatic pathways could suggest active matrix remodeling and fibrosis, as was also observed by histology (Figure 1C). This is consistent with reports that the IFP can drive fibrotic changes. For example, Bastiaansen-Jenniskens et al. showed that IFP fat-conditioned medium stimulates synoviocyte proliferation and collagen production.^51^ Moreover, *in vivo* studies show that IFP fibrosis is linked to increased immune infiltration and CCL2 and TGFβ levels,^52^ suggesting a possible connection with the observed upregulation of metabolic pathways and immune cell persistence in our study. Together, this data supports a model in which the elevated metabolic and enzymatic capacity of OA IFP, revealed by our proteomics, could be a mechanistic contributor to the fibrotic remodeling seen in advanced disease. However, the results could also reflect the age differences between patients with OA and preOA.

Neutrophils have recently been reported to contribute to the changes in the extracellular matrix and fibrosis.^6^^,^^53^ Furthermore, neutrophils and the content of their granules have also been described as proangiogenic.^29^^,^^54^^,^^55^ Consistently, we observed increased vasculature and fibrosis in OA IFP (Figures 1A–1E), both of which are recognized as important in IFP OA pathogenesis.^17^^,^^56^ Specifically, increased fibrosis has been suggested to decrease IFP shock-absorbing capacity and change its biomechanical properties, thus promoting joint damage.^17^^,^^21^^,^^57^ Our data imply a maladaptive microenvironment in OA IFP. We hypothesize that this microenvironment could influence neutrophil phenotype and support fibrotic and angiogenic remodeling, potentially exacerbating joint damage and OA progression; however, these associations remain correlative.

### Limitations of the study

Neutrophils are notoriously challenging to study due to their sensitivity to various stimuli that cause rapid degranulation and subsequent cell death. Furthermore, their low RNA content and high transcriptional noise, especially in their mature tissue resident state, also make transcriptomic analysis difficult.^58^^,^^59^ Additionally, reliable markers to identify mature neutrophil subtypes remain poorly defined. This makes it difficult to conclusively assign neutrophil function or lineage. This limits our ability to isolate and functionally validate distinct neutrophil subsets and leads the study to remain largely descriptive. Furthermore, the demographic differences between patients with OA and preOA, donor-to-donor variability, small sample sizes, and the small tissue pieces received for preOA make comparing neutrophil behavior between the two groups challenging. Specifically, advanced age has been shown to affect adipocyte size and collagen composition even in non-OA individuals; these age-related changes could affect our proteomic results regardless of disease state.^60^^,^^61^

Despite these challenges, our study reveals that neutrophils in OA IFP acquire a tissue-specific phenotype, marked by the expression of antigen presentation machinery. This finding identifies IFP and neutrophils as potential contributors to the OA pathogenesis. The study reiterates the importance of considering tissue-resident neutrophils and the IFP in the context of knee OA pathology and progression. We hope to inspire further research focusing on evaluating the role of IFP neutrophils in OA progression and their interaction with other immune and stromal cells, such as macrophages and adipocytes, particularly in relation to antigen presentation, and therapeutic targeting.

## Resource availability

### Lead contact

Further information and requests for resources and reagents should be directed to and will be fulfilled by the Lead Contact, Marcy Zenobi Wong (marcy.zenobi@hest.ethz.ch).

### Materials availability

This study did not generate new unique reagents.

### Data and code availability

• Data have been deposited at ETH Zurich Research Collection 2025 and are publicly available as of the date of publication. The mass spectrometry proteomics data have been deposited to the ProteomeXchange Consortium via the PRIDE partner repository and are publicly available as of the date of publication. Accession numbers are listed in the key resources table.
• This article does not report original code.
• Any additional information required to reanalyze the data reported in this article is available from the lead contact upon request.

## Acknowledgments

We would like to thank the ScopeM center and the Flow Cytometry Core Facility at ETH Zurich for providing technical support. We would specifically like to thank Renan Antonialli and Irini Vgenopoulou for their help with cell sorting.

This work was supported by the European Union's HORIZON EUROPE 2021-2027 Research and Innovation Actions (grant no. 101095084) and by the Swiss State Secretariat for Education, Research and Innovation (SERI) under contract number 22.00462.

## Author contributions

K.B., I.A.W., M.Z.W., and F.M. contributed to the research design; K.B. and S.Z. conducted the research; K.B., I.B., and A.P. analyzed the data; K.B. conceived the project, K.B. wrote the article, M.Z.W. and I.A.W. supervised the project. All authors contributed to the interpretation of the results, critical revision of the article, and have given their final approval of the article.

## Declaration of interests

The authors declare no competing interests.

## STAR★Methods

### Key resources table

**Table:** 

REAGENT or RESOURCE	SOURCE	IDENTIFIER
**Antibodies**		

Anti human CD8-BUV395	BD Horizon	Cat#56795; RRID: AB_3720784
Anti human CD3-BUV496	BD Horizon	Cat#612940; RRID: AB_2870222
Anti human CD56- BUV563	BD Horizon	Cat #612929; RRID: AB_2916880
Anti human CD16- BUV615	BD OptiBuild	Cat #751572; RRID: AB_2875567
Anti human PD1- BUV661	BD OptiBuild	Cat #750260; RRID: AB_2874457
Anti human CD80- BUV737	Invitrogen	Cat #367-0809-42; RRID: AB_2895983
Anti human CD45- BUV805	BD Horizon	Cat #612892; RRID: AB_2870180
Anti human CD25- BV421	BioLegend	Cat #356113; RRID: AB_2562163
Anti human CD123- BV480	BD Horizon	Cat #566133; RRID: AB_2739532
Anti human CD90- BV510	BioLegend	Cat #328126; RRID: AB_2563850
Anti human CD11b- BV570	BioLegend	Cat #301325; RRID: AB_11150781
Anti human CD105- BV605	BD OptiBuild	Cat #752991; RRID: AB_2917945
Anti human Nkp46- BV650	BioLegend	Cat #331927; RRID: AB_2562442
Anti human CD73-BV711	BioLegend	Cat #344026; RRID: AB_2687232
Anti human CD15-BV750	BD OptiBuild	Cat #747426; RRID: AB_2872112
Anti human CD206-BV785	BioLegend	Cat #321142; RRID: AB_2734302
Anti human CD33-FITC	BioLegend	Cat #366620; RRID: AB_2566422
Anti human CD14-SparkBlue550	BioLegend	Cat #367147; RRID: AB_2820021
Anti human CD141-BB700	BD OptiBuild	Cat #742245; RRID: AB_2740668
Anti human CD117-PE	BioLegend	Cat #313204; RRID: AB_314983
Anti human CD34-PE CF594	BD Horizon	Cat #562383; RRID: AB_11154586
Anti human CD19-PE Fire640	BioLegend	Cat #302273; RRID: AB_2860772
Anti human CD127-PE Cy7	BioLegend	Cat #986008; RRID: AB_2924646
Anti human CD86-AF647	BioLegend	Cat #305415; RRID: AB_528882
Anti human CD11c-R718	BD Horizon	Cat #566932; RRID: AB_2869954
Anti human HLADR-APC H7	BD Pharmigen	Cat #561358; RRID: AB_10611876
Anti human CD4-APC Fire810	BioLegend	Cat #344661; RRID: AB_2860883
Anti human CD74-FITC	BioLegend	Cat #344026; RRID: AB_2687232
Anti human Fc Block	BD Pharmingen	Cat #564219; RRID: AB_2728082
Rabbit IgG anti CD15	abcam	Cat #ab218403; RRID: AB_3720782
Mouse IgG anti CD16	BioLegend	Cat #302049; RRID: AB_2783156
Mouse IgG anti HLADR	abcam	Cat #ab20181; RRID: AB_445401
Mouse IgG anti NE	Santa Cruz	Cat #sc-55549; RRID: AB_831596
Rabbit IgG anti CD3	abcam	Cat # ab5690; RRID: AB_305055

**Biological samples**		

Human OA and preOA IFP tissue	Schulthess Klinik, Zurich, Switzerland	NA

**Chemicals, peptides, and recombinant proteins**		

Collagenase Type I	Merck	Cat # C1-BIOC
DNase I	Stemcell	Cat # 100-0683
CellTrace™ CFSE Cell Proliferation Kit	Thermo Fisher	Cat # C34554

**Critical commercial assays**		

Pierce™ Bradford Protein Assay Kit	ThermoFisher Scientific	Cat #23200
Zombie NIR fixable viability kit	BioLegend	Cat #423105c

**Deposited data**		

Raw and analyzed data	This paper	https://doi.org/10.3929/ethz-c-000783053
Proteomic data	This paper	PRIDE PXD073289

**Software and algorithms**		

clusterProfiler	Yu et al.^34^	RRID:SCR_016884
fgsea	Korotkevich et al.^35^	RRID:SCR_020938
Spectre v1.0.0 R Package	Ashhurst et al.^37^	RRID:SCR_000266
FlowSOM	Van Gassen et al.^38^	RRID:SCR_016899
FlowJo 10	BD	RRID:SCR_008520
GraphPad Prism 10.4.0.	GraphPad	RRID:SCR_002798

### Experimental model and study participant details

#### Study participants

Ethical approval for the study was granted by the Cantonal Ethical Commission of Zurich (Kantonale Ethikkommission Zürich, BASEC number 2021-0186). Written informed consent was obtained from all participants before the surgery, in accordance with the Declaration of Helsinki.

Human IFP samples from end-stage OA patients undergoing total knee arthroplasty (termed OA) and patients undergoing knee surgery to treat non-traumatic cartilage defects (termed preOA) (Table 1) were collected. Participants were recruited based on the following criteria: for OA, patients with knee OA diagnosis but no rheumatoid arthritis (RA) diagnosis undergoing total knee arthroplasty. For preOA, a degenerative cartilage defect which required surgical intervention with no diagnosis of OA or RA. Participants were excluded if they received treatment elsewhere at the same time, had an infection or had any non-rheumatic systemic inflammatory disease. The included samples were subjected to different analyses described in the STAR Methods, where CD45^+^ cells correspond to all immune cells (Table 1). Human healthy blood samples were obtained from Blutspende Zurich, with anonymised information.

### Method details

#### Proteomics

IFP samples were selected and cut into pieces (Table 1). The samples were then submerged in 300 uL of RIPA buffer per 100 mg of tissue and sonicated on ice until the tissue was homogenised. Amplitude of 60 was used in intervals to not overheat the sample. For protein measurement, the samples were first 10x diluted in PBS, as RIPA buffer interferes with the measurement. Protein concentration (Table 1) was measured with the Pierce™ Bradford Protein Assay Kit (Thermo Fischer, USA) according to kit instructions.

Proteomic analysis was performed using high-resolution micro–liquid chromatography coupled to tandem mass spectrometry (micro-LC–MS/MS) on a hybrid quadrupole TripleTOF 6600 system (Sciex, USA).

Protein identification was carried out using a qualitative data-dependent acquisition (DDA) method, while relative protein quantification was achieved through data-independent acquisition using the sequential window acquisition of all theoretical mass spectra (SWATH) approach. Protein identification and quantification were filtered using a false discovery rate (FDR) threshold of 5% and a p-value ≤ 0.05.

Differential expression analysis between OA and pre-OA samples was conducted in R (version 4.2), and proteins with a p-value ≤ 0.05 were considered significantly regulated. Functional enrichment analysis of differentially expressed proteins was performed using Gene Ontology (GO) terms for Biological Process and Molecular Function categories with the clusterProfiler^62^ and fgsea^63^ R packages.

#### Histology

The IFP samples were dissected to remove visible synovial membrane or attached cartilage pieces and fixed in 4% paraformaldehyde and embedded in paraffin. Serial 5 μm thick sections were cut using a microtome (HM 325, Microm, GER). Cut sections were rehydrated for histological staining with Hematoxylin & Eosin (H&E), Masson’s trichrome and immunofluorescent staining. For H&E Staining rehydrated IFP sections were stained with Gill No. 3 hematoxylin (Merck, GER) for 5 min at room temperature, washed with water 10 min and then submerged in a blueing solution (0.1% Na2CO3) for 40 seconds, followed by counterstaining in 0.25% Eosin Y solution for 1 minute. After washing in 100% EtOH, the sections were dehydrated in Xylene and coverslipped using Eukitt mounting medium.

The H&E stained samples were scored by three blinded and independent scorers according to the histological scoring protocol provided in Table 2, adapted from the rat scoring protocol proposed by Kitagawa et al.^64^

#### Immunofluorescence staining

Rehydrated IFP sections were boiled in sodium citrate buffer (pH 6) for 1 hour for antigen retrieval followed by blocking with 5% BSA for 1 hour. After they were incubated overnight at 4°C with primary antibodies against CD15 (FUT4/1478R, Abcam, UK, 1:100, 2 μg/mL), NE (sc-55549, Santa Cruz, USA, 1:50, 4 μg/mL), HLA-DR (EPR3692, Abcam, UK, 1:1000, 0,8 μg/mL) and CD74 conjugated with FITC (555540, BD Biosciences, USA, 1:50) diluted in 1% BSA. The next day after washing in PBS, the sections were incubated with secondary antibodies anti-mouse AF647 (A-21240, Invitrogen, USA, 1:500, 4 μg/mL), anti-mouse AF568 (A-11004, Invitrogen, USA, 1:500, 4 μg/mL) and anti-rabbit AF555 (A21428, Invitrogen, USA, 1:500, 4 μg/mL) and DAPI (Thermo-Fisher, USA, 1:1000) diluted in 1% BSA. Stained sections were coverslipped with Anti-Fade Fluorescence Mounting Medium (Abcam, UK) and imaged within 24 hours with a confocal microscope (Confocal Leica SP8-AOBS-CARS) with 63x magnification and then stored at -20°C for future use. The images were processed and analysed with Fiji v.2.16.0.

#### Sample processing

IFP samples collected at the hospital were transferred to the laboratory in 50 mL tubes containing 20 mL of RPMI media (11875093, Gibco, USA) at room temperature. The samples were dissected to separate visible synovial membrane from the adipose tissue which was collected and cut into smaller pieces. The pieces were transferred into C tubes (Miltenyi, GER) with 4 mL of tissue dissociation buffer per 1 g of tissue. The tissue dissociation buffer contains RPMI media (11875093, Gibco, USA) supplemented with 500 U/mL Collagenase Type I (C1-BIOC, Merck, USA), 200 U/mL DNase I (# 100-0683, Stemcell, CAN) and 10 mM CaCl_2_. The tissue was homogenised directly after with the Gentle MACS Dissociator (Miltenyi, GER) with the program mr_adipose_1 at room temperature. After homogenisation the tubes were transferred to an incubator at 37°C for the tissue to digest for 25 minutes. After digestion the tubes were transferred to a sterile laminar flow hood and the tissue digest was triturated through 70 μm filters (Miltenyi, GER), washed with RPMI media containing 200 U/mL DNase I and centrifuged at 500*g* for 10 minutes. The pellets were sequentially lysed with 1x red blood cell lysis buffer (BioLegend, USA), washed with phosphate-buffered saline (PBS) (pH 7.4) buffer and centrifuged at 500*g* for 10 minutes until the supernatant and pellet were both free from erythrocytes.

Synovial fluid samples collected from matched OA donors were diluted 1:1 with wash buffer containing RPMI media and 200 U/mL DNase I and centrifuged at 500*g* for 10 minutes. The pellet was then lysed with 1x red blood cell lysis buffer, washed with phosphate-buffered saline (PBS) (pH 7.4) buffer and centrifuged until the supernatant and pellet were both free from erythrocytes.

Healthy blood samples were sequentially lysed with 1x red blood cell lysis buffer, washed with phosphate-buffered saline (PBS) (pH 7.4) buffer and centrifuged at 500*g* for 10 minutes until the supernatant and pellet were both free from erythrocytes.

#### Flow cytometry and sorting

Cell pellets were washed and resuspended in FACS buffer (0,5% heat inactivated BSA in PBS, 200 U/mL DNase I, 2 mM EDTA). The cells were first incubated for 10 minutes with human Fc block (564219, BD Biosciences, USA,1:50) to reduce non-specific antibody binding. After washing they were incubated for 30 minutes with the respective antibody panel (Table S2). The cells intended for flow cytometry analysis were then incubated for 10 minutes with Zombie NIR^TM^ fixable viability kit (#423105c, BioLegend, USA, 1:1000) to identify viable cells. They were subsequently fixed in 4% paraformaldehyde for 10 minutes and analysed on a 5-laser spectral flow cytometer (Aurora from Cytek Biosciences, USA). The cells intended for sorting were stained with Sytox blue (#S34857, Thermo Fisher, USA) right before the sort to assess viability and sorted with a 7-laser spectral sorter (Bigfoot from Thermo Fisher, USA). Viability of sorted neutrophils exceeded 95% as assessed by post sort analysis (Table S3). Data analysis was performed with FlowJo 10 (BD Biosciences). For more detailed analysis we imported data in R v4.3.0 and analysed it with a custom script based on Spectre v1.0.0 R Package.^65^ For the analysis we proceeded with live cells and removed outliers based on 0.01 quantile. Afterwards data was normalized with 5000 scale factor following by FlowSOM^35^ iterative clustering by keeping the same number of cells from each condition and compared abundances for the clusters between conditions using Mann Whitney test.

#### Cell culture

Donor matched CD3^+^ T cells and HLA-DR+ neutrophils were sorted from IFP of 4 OA donors as well as donor matched CD3^+^ T cells and HLA-DR- neutrophils from blood of 4 healthy donors (Figure S2). Neutrophils were resuspended in unsupplemented AIM V™ media (12055091, Gibco, USA) immediately after sorting. T cells were first stained with CellTrace™ CFSE Cell Proliferation Kit (C34554, Thermo Fisher, USA) according to manufacturer’s instructions before being resuspended in the same media. An aliquot of stained T cells was used to validate the CFSE staining and then remaining T cells were incubated for 5 days either as a monoculture, mixed 4:1 with HLA-DR^-^ neutrophils or mixed 4:1 with HLA-DR^+^ neutrophils in a donor matched manner in 96 well v bottom plates. After 5 days the cells were analysed by flow cytometry.

### Quantification and statistical analysis

Statistical significance between two groups was assessed using the Mann–Whitney test. When there were more than two groups, unpaired one-way analysis of variance (ANOVA) with Tukey’s correction was used. Data is plotted to represent the mean with 95% confidence intervals (CI). Significance is indicated with asterisks: ∗ p < 0.05, ∗∗ p < 0.01, ∗∗∗ p < 0.001, ∗∗∗∗ p < 0.0001, ns = not significant. Statistical analyses were conducted using GraphPad Prism 10.4.0.
