# Supplementary material for: Enrichment of HLA-DR+ neutrophils in osteoarthritic infrapatellar fat pad
Source: iScience. 2026 Mar 5;29(4):115214. doi: 10.1016/j.isci.2026.115214 (PMC12990351; doi:10.1016/j.isci.2026.115214)
Supplement: Document S1. Figures S1–S7 and Tables S1–S3 [file mmc1.pdf]

iScience, Volume *29*

Supplemental information

Enrichment of HLA-DR$^+$ neutrophils in osteoarthritic infrapatellar fat pad

Kajetana Bevc, Shipin Zhang, Andres Pazos, Ivan Berest, Marina Fonti, Gian Salzmann, Valentino Bruhin, Jakob Hax, Ana Alonso Perez, Rodolfo Gomez, Florian Mair, Isabelle C. Arnold, and Marcy Zenobi Wong

Supplementary material

Supplemental figures and legends

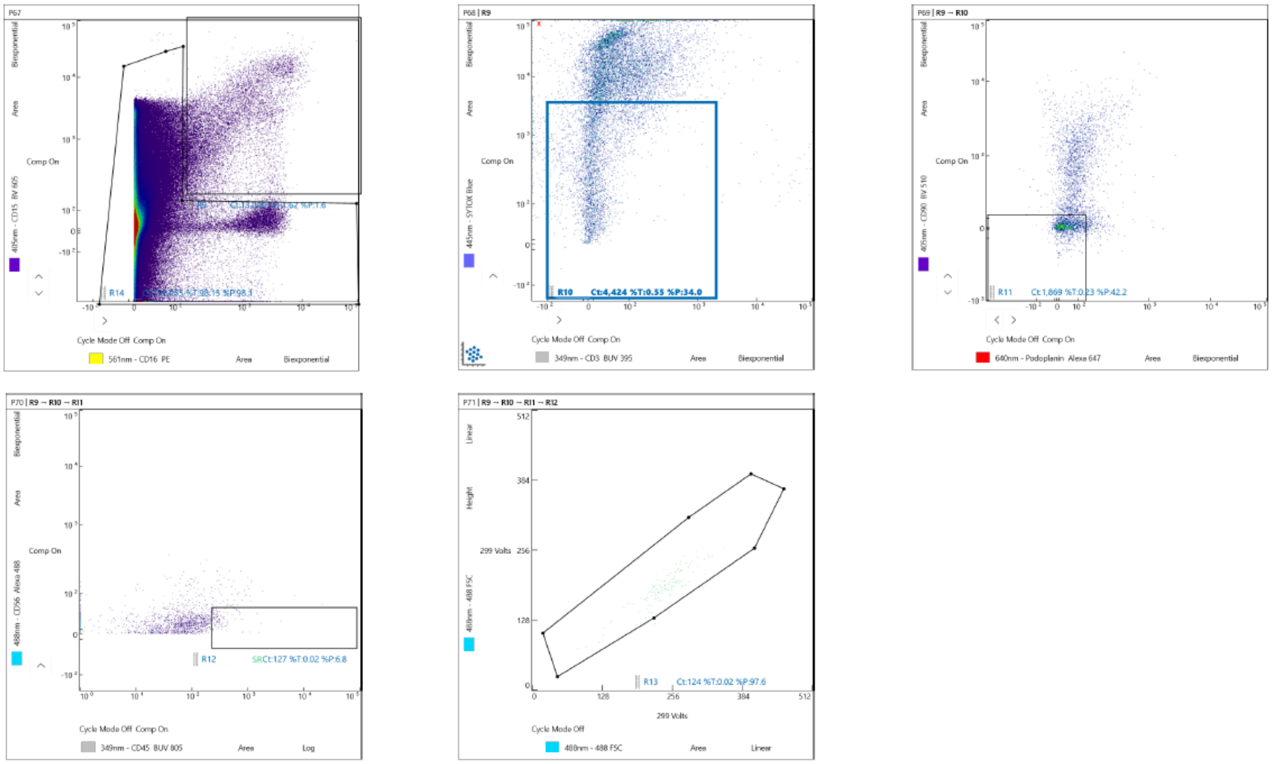

Figure S1 Neutrophil enrichment sorting strategy

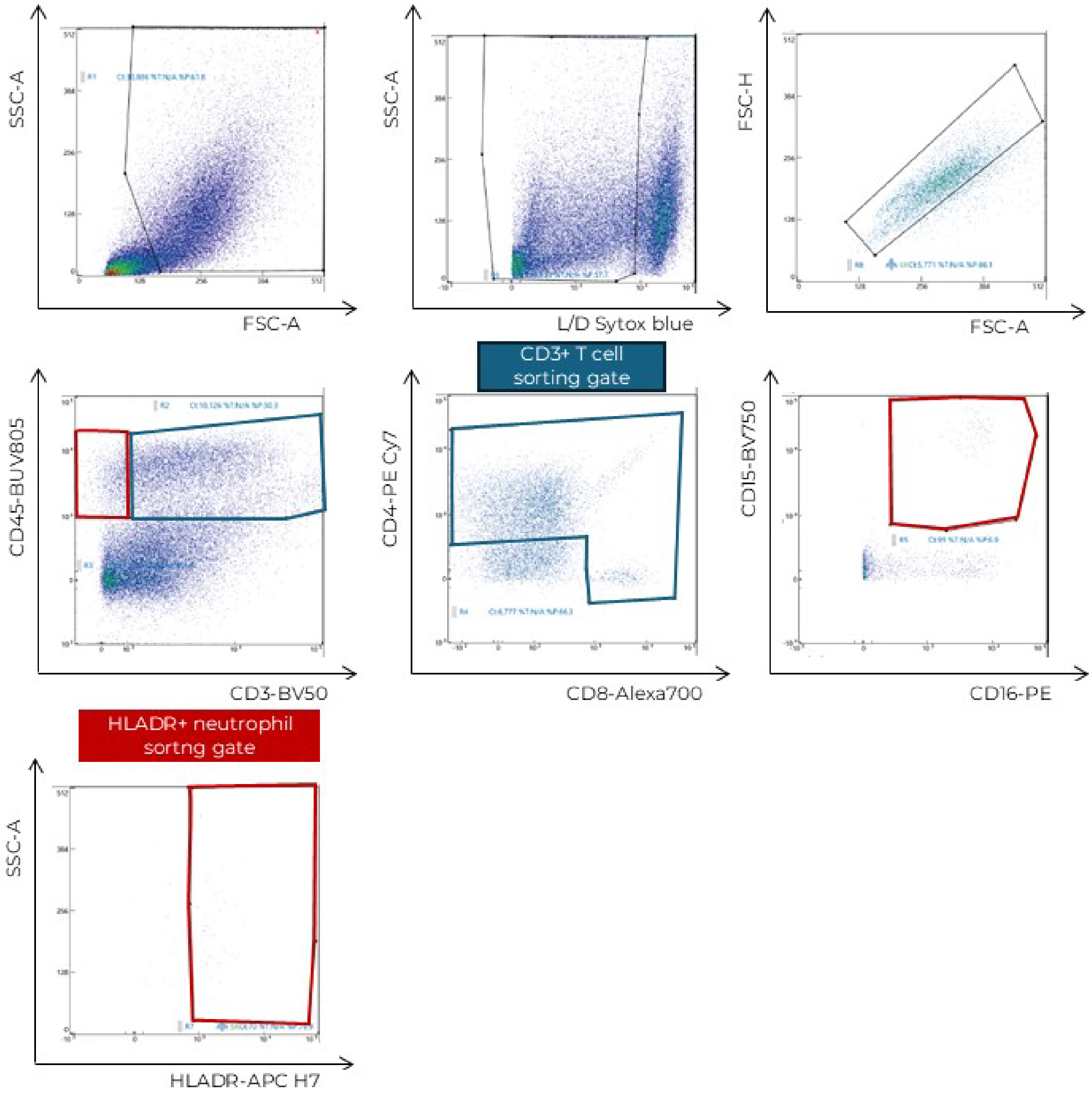

Figure S2 HLADR+, HLADR- and CD3+ T cell sorting strategy

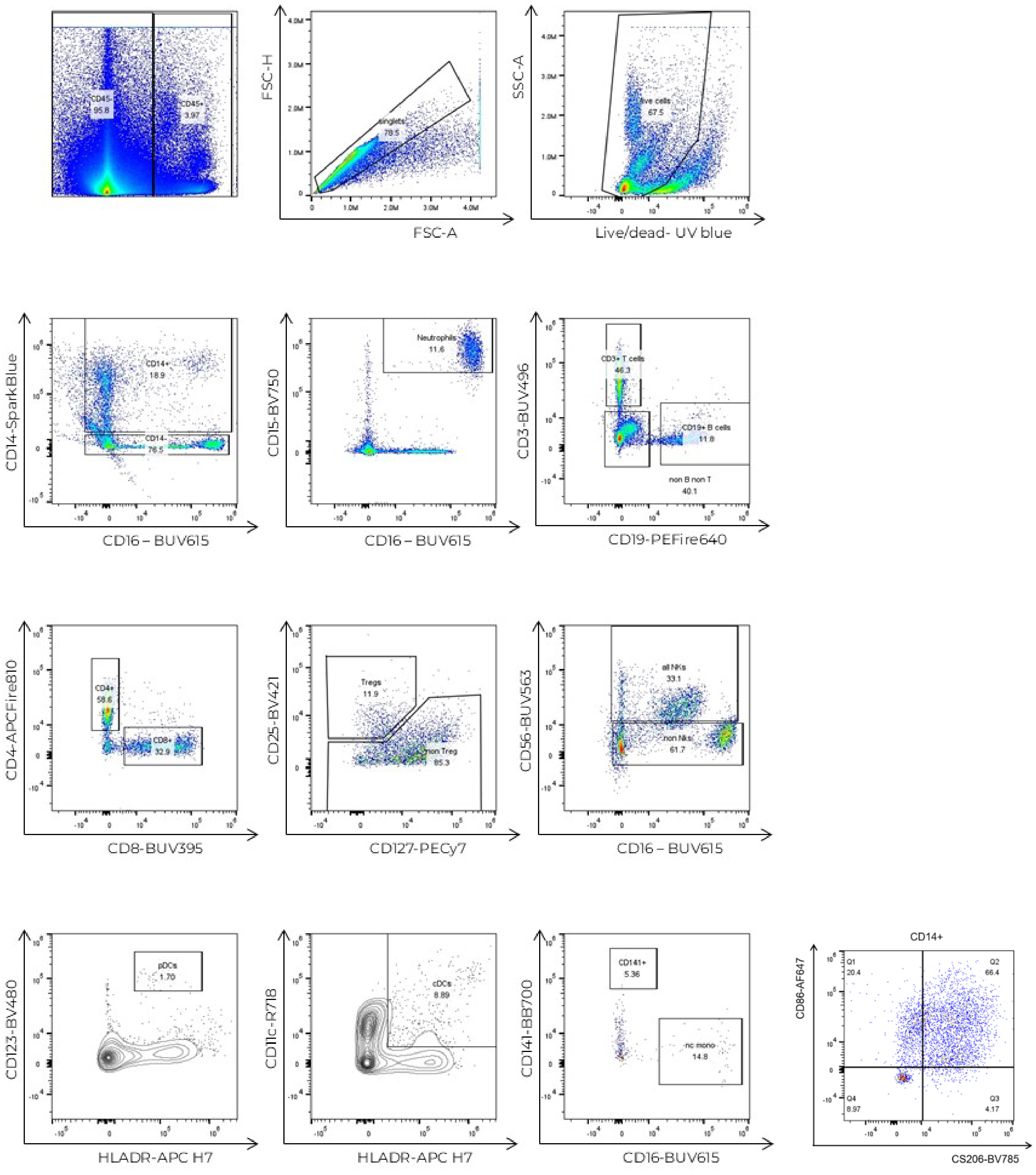

Figure S3 Gating strategy for the stromal vascular fraction of OA vs preOA IFP

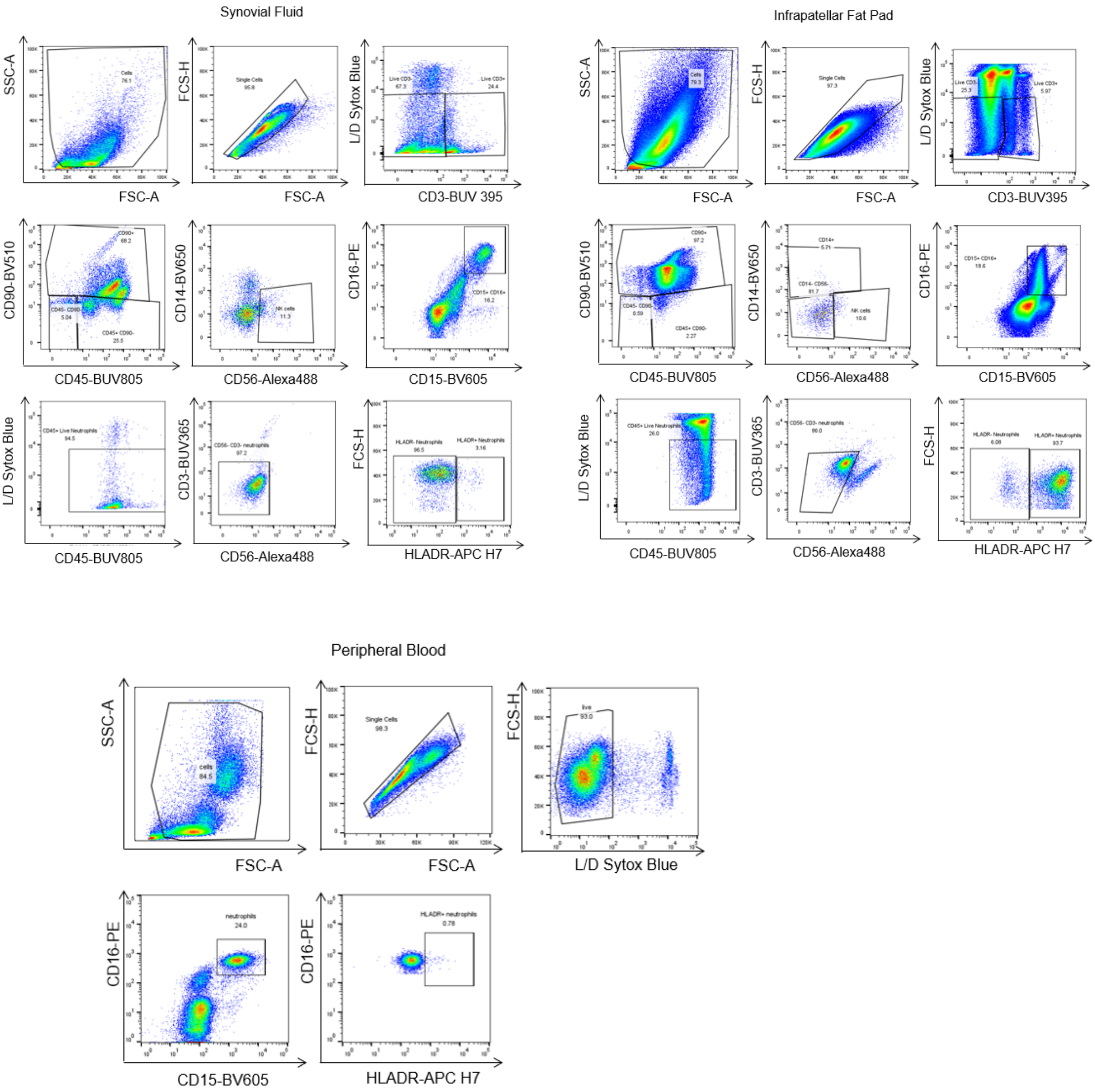

Figure S4 HLADR Neutrophil analysis gating strategies on matched OA synovial fluid and infrapatellar fat pad and healthy blood

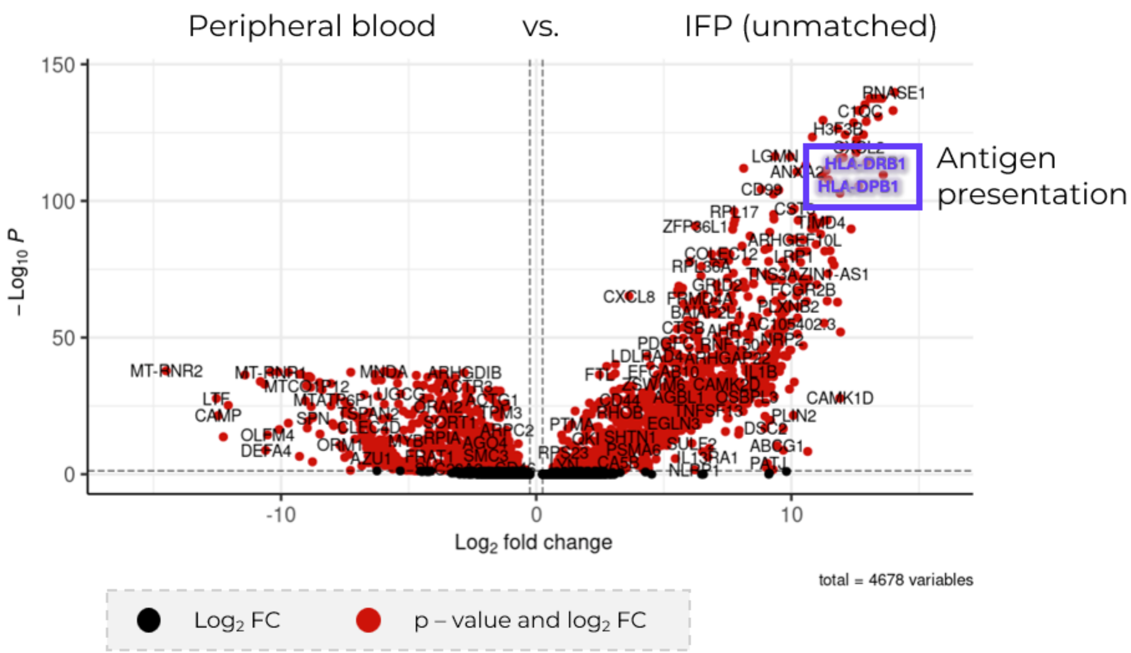

Figure S5 Differential gene expression between healthy human peripheral blood (publicly available dataset) and cluster 8 from OA IFP

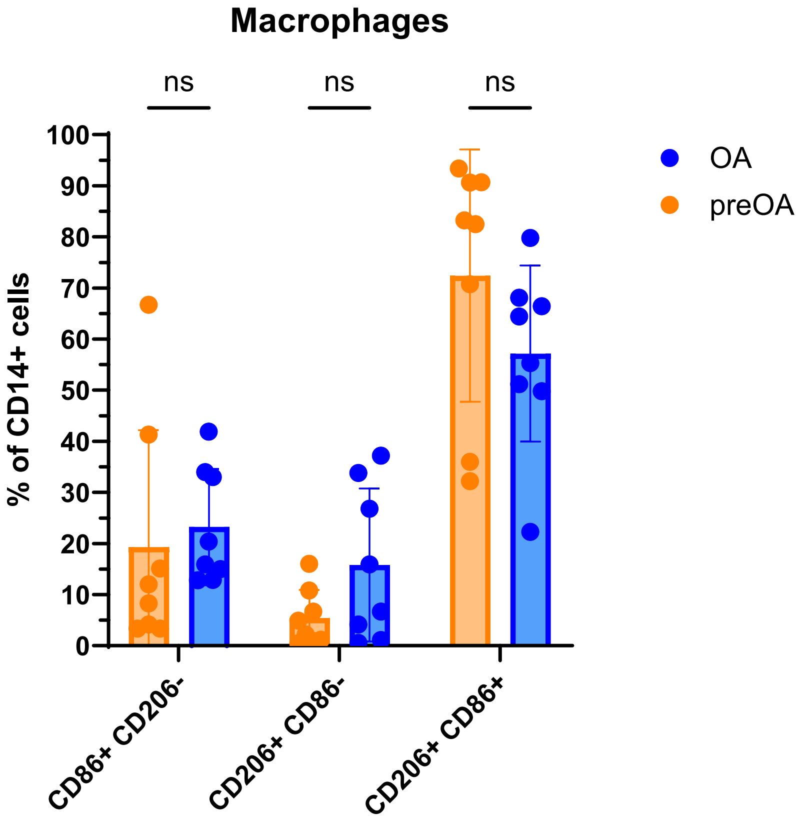

Figure S6 Macrophage polarisation in OA IFP compared to preOA IFP

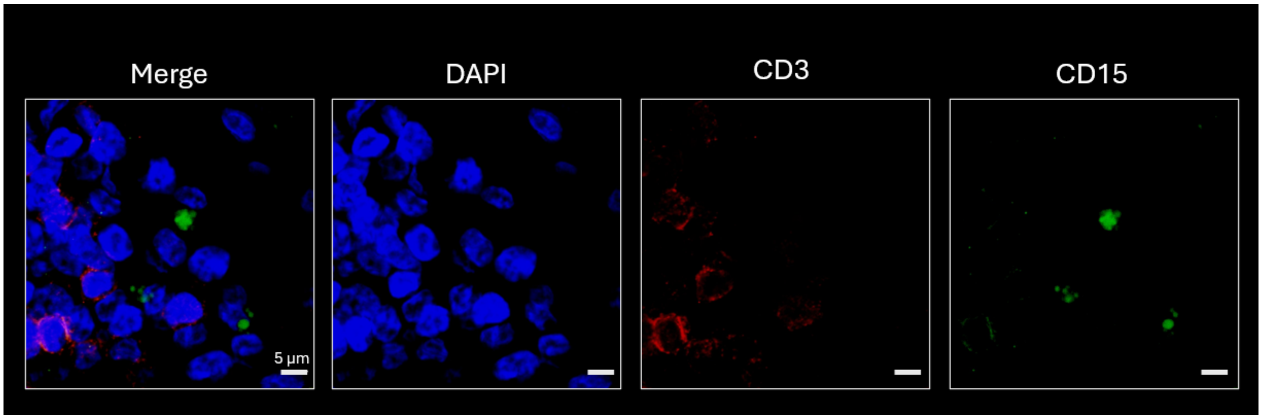

Figure S7 Immunofluorescence staining of OA IFP T cells (CD3, red) and neutrophils (CD15, green), scale bars correspond to 5 µm

Supplemental tables

Table S1 List of differentially expressed proteins in OA and preOA

OA enriched

Symbol	OA Mean	preOA Mean	log2FoldChange	pvalue
KIF15	2384,421667	1070,483333	1,155377054	0,023856623
PMS1	6176,3	1634,448333	1,917939057	0,028460698
C5orf22	2011,245	882,4583333	1,188488768	0,028798587
TCEA3	5591,25	2518,723333	1,150478194	0,033833881
CHST3	1039,411667	311,3133333	1,739327885	0,047630298

preOA enriched

Symbol	OA Mean	preOA Mean	log2FoldChange	pvalue
MEA1	2107,55	5783,75	-1,456438335	0,001442646
HADHA	1863,516667	5737,266667	-1,622335854	0,002937491
FAM151A	1910,3	4066,566667	-1,090012044	0,005178954
TUBA8	2708,78	9506,483333	-1,811268527	0,015418688
TAF4	1284,896667	3216,816667	-1,323981376	0,018037003
FAM129B	873,7566667	1773,63	-1,021401615	0,020540107
ATP5J2	2555,633333	9175,066667	-1,844037779	0,019668347
C9orf114	527,6183333	3139,073333	-2,572772132	0,019376439
HIP1R	1066,288333	4367,966667	-2,034364239	0,020497402
PDAP1	246,58	2681,91	-3,443133131	0,02847148
HIST1H1C	2262,54	7491,383333	-1,727288848	0,024080391
KIAA1211	1237,091667	3265,15	-1,400196864	0,023416956
PF4	2935,785	5934,633333	-1,015412579	0,023933051
DFFA	414,62	5241,783333	-3,660196107	0,032187694
INPP5F	551,635	3388,028333	-2,618660039	0,028393377
PLEKHF2	1165,811667	4629,836667	-1,989626554	0,032477204
CSNK1E	1119,296667	3263,416667	-1,54379073	0,037044041
PRPF38B	2611,493333	5559,533333	-1,090088768	0,028712862
FAM213A	2498,116667	15553,45	-2,638321868	0,037332202
PRKAB2	711,2133333	3582,076667	-2,332441939	0,038937963
SOD2	2409,983333	9793,933333	-2,022865206	0,033912413
POGLUT1	1025,028333	3093,361667	-1,59351173	0,044346664
CPNE8	1832,465	3799,016667	-1,051840398	0,036177949
UBE2E2	197,5583333	1778,895	-3,17063075	0,03991477
RPL4	600,7016667	3454,2	-2,523631046	0,040426721
HNRNPC	160,5616667	769,555	-2,260896942	0,039071861
HIST1H1D	1296,883333	5551,25	-2,097763965	0,039521345
SNAP29	1730,375	3873,533333	-1,162565428	0,040092946
ARMC10	1172,808333	2506,283333	-1,09558226	0,038773997
SNRPD2	781,7866667	2595,205	-1,731001619	0,042387469
VWA9	583,0583333	1684,883333	-1,530936565	0,047294786

IRF5	2321,166667	5316,416667	-1,195604062	0,042173831
SREK1	784,8466667	4381,96	-2,481093584	0,045733696
AURKB	998,6133333	4277,43	-2,098746171	0,049877899
FAM162A	944,6666667	2994,465	-1,664421012	0,048039337
SENP3	2218,283333	4884,016667	-1,138624476	0,048779055

Table S2 Antibody panel

Company and catalogue number	Antigen	Flourophore	Dilutions
BD Horizon #563795	CD8	BUV395	1: 80
BD Horizon #612940	CD3	BUV496	1: 20
BD Horizon #612929	CD56	BUV563	1: 80
BD OptiBuild #751572	CD16	BUV615	1: 160
BD OptiBuild #750260	PD1	BUV661	1: 80
Invitrogen #367-0809-42	CD80	BUV737	1: 40
BD Horizon #612892	CD45	BUV805	1: 40
BioLegend #356113	CD25	BV421	1: 10
BD Horizon #566133	CD123	BV480	1: 80
BioLegend #328126	CD90	BV510	1: 40
BioLegend #301325	CD11b	BV570	1: 80
BD OptiBuild #752991	CD105	BV605	1: 40
BioLegend #331927	Nkp46	BV650	1: 40
BioLegend #344026	CD73	BV711	1: 80
BD OptiBuild #747426	CD15	BV750	1: 20
BioLegend #321142	CD206	BV785	1: 20
BioLegend #366620	CD33	FITC	1: 100
BioLegend #367147	CD14	SparkBlue550	1: 80
BD OptiBuild #742245	CD141	BB700	1: 40
BioLegend #313204	CD117	PE	1: 25
BD Horizon #562383	CD34	PE-CF594	1: 10
BioLegend #302273	CD19	PE-Fire640	1: 40
BioLegend #986008	CD127	PE-Cy7	1: 40
BioLegend #305415	CD86	AF647	1: 40
BD Horizon #566932	CD11c	R718	1: 80
BD Pharmingen #561358	HLA-DR	APC-H7	1: 40
BioLegend #344661	CD4	APC-Fire810	1: 100

Table S3 Blood neutrophil viability post-sorting and culturing

Post-sort Viability	D1	D2	D3
Immediately %Live	98,4	99,4	99,9
1 hr 30 min %Live	98,6	99,0	99,7
24 hr %Live	96,6	95,8	96,1
